# Pharmacological effects of ginseng and ginsenosides on intestinal inflammation and the immune system

**DOI:** 10.3389/fimmu.2024.1353614

**Published:** 2024-04-18

**Authors:** Linxian Zhao, Tongbo Zhang, Kai Zhang

**Affiliations:** Department of General Surgery, The Second Hospital of Jilin University, Jilin University, Changchun, Jilin, China

**Keywords:** ginseng, ginsenosides, intestinal system diseases, intestinal immune, intestinal inflammation

## Abstract

Intestinal inflammatory imbalance and immune dysfunction may lead to a spectrum of intestinal diseases, such as inflammatory bowel disease (IBD) and gastrointestinal tumors. As the king of herbs, ginseng has exerted a wide range of pharmacological effects in various diseases. Especially, it has been shown that ginseng and ginsenosides have strong immunomodulatory and anti-inflammatory abilities in intestinal system. In this review, we summarized how ginseng and various extracts influence intestinal inflammation and immune function, including regulating the immune balance, modulating the expression of inflammatory mediators and cytokines, promoting intestinal mucosal wound healing, preventing colitis-associated colorectal cancer, recovering gut microbiota and metabolism imbalance, alleviating antibiotic-induced diarrhea, and relieving the symptoms of irritable bowel syndrome. In addition, the specific experimental methods and key control mechanisms are also briefly described.

## 1 Introduction

The dysregulation of the intestinal inflammations system and adaptive immune imbalance can result in a series of intestinal diseases and diseases in distant body sites, such as inflammatory bowel disease (IBD), irritable bowel syndrome (IBS), intestinal infectious diseases, intestinal system tumor, and neurological disease (1, 2). Among them, IBD is the most prevalent disease, the incidence of which is significantly increasing and continues to rise during the twentieth century (3). Despite multiple anti-inflammation and immunomodulating agents have been used for intestinal system diseases treatment, limited efficacy and serious side effects remain major clinical challenges. With more and more immunomodulatory and anti-inflammatory pharmacological effects having been developed, traditional Chinese medicines have been used for various inflammation and immune disorders treatment. As one of the most well-known traditional Chinese medicines, ginseng has a long history as an herbal medicine for various disease (4), especially, which possessed stronger anti-inflammatory and immunoregulation effects. In the past decades, an increasing number of studies have indicated that ginseng and its major constituent, various ginsenosides, were capable of effectively relieving the severity of gastrointestinal colitis in animal models (5). In this review, we mainly focus on how ginseng and ginsenoside regulate intestinal system inflammation and the immune homeostasis.

## 2 Intestinal inflammations

Under normal physiological conditions, inflammation response is a self-resolving and self-protective process, through which the body attempts to counteract tissue injury damage or infection and together remove pathogens and cell debris (6). However, continuous inflammation response is harmful to the host. Therefore, once the harmful stimuli has been eliminated, the related proinflammatory response should be curtailed immediately (7). Intestinal system inflammations represent a group of relapsing and multifactorial disorder associated with physical and mental well-being, mainly including intestinal tumor and inflammatory bowel disease (IBD) such as ulcerative colitis (UC), irritable bowel disease (IBS), and Crohn’s disease (CD). According to the latest epidemiological data from the World Health Organization, IBD has become a global burden, and the incidence are rising globally, especially in newly industrialized regions of North America and Europe (8–10).

To date, multiple factors have been verified to be involved in the regulation of IBD, such as gut microbiota, gut mucosal inflammation, cell immune response, oxidative stress, and eating habits (11). The normal intestinal epithelial barrier is composed of the mucosa, the glycocalyx, and tight junctions, all of which are involved in maintaining gut barrier integrity and intestinal homeostasis (12). The intestinal tight junction proteins form a physical barrier and the commensal microbiome can further reinforce intestinal barrier homeostasis through preserving gastrointestinal physiology and intestinal immune system development (13, 14). The dysfunction of IBD is primarily associated with intestinal mucosal inflammation. Inflammation responses are a complex process involving a series of cellular and molecular changes, including altering vascular permeability, promoting aggregation of leukocyte, and regulating expression levels of inflammatory cytokines (15). Cytokines play a crucial role in the pathogenesis of IBD. In terms of UC and CD, it was found that the proinflammatory cytokines such as IL-1, TNF-α, IL-1β, and IL-6 were significantly upregulated in the inflamed intestine, thus further changing the composition of the tight junction microenvironment (16). In addition, gut microbiome composition and mucosal immune responses both play an important role in intestinal barrier homeostasis (17). A recent study by Khan and co-workers suggested that dysregulated gut microbiota was able to specifically activate T-helper cell immune response, which can result in further dysregulation of intestinal barrier function and a sustained inflammatory response (18).

## 3 Intestinal immune

The immune system plays an important role in the human body against foreign bacteria and virus infections. The human immune system consists of primary and secondary lymphoid systems (19). Mucosal-associated lymphoid tissue is one of the most typical secondary lymphoid systems, which works as a physical and immunological barrier (20). As the largest mucosal-associated lymphoid tissue, gut-associated lymphoid tissue is very essential for the maintenance of intestinal homeostasis, the components of which mainly include Peyer’s patches and mesenteric lymph nodes (21). Intestinal immune dysfunction also has been reckoned as a major pathogenesis of IBD (22). As the primary site of gut microbiota colonization, more than trillions of microorganisms have been discovered on the surfaces of the ileum and colon, mainly including fungi, protozoa, viruses, archaea, and predominantly bacteria (23). It has been reported that gut microbiota was capable of regulating the innate immune system and intestinal immune homeostasis (24). For example, *Clostridium difficile* is capable of promoting goblet cells and dendritic cells secreting cytokines (TGF-β and IL-10), thus further generating ample signals to upregulate Treg population (25). In addition, some important components such as vitamin K and SCFA are produced by intestinal microbiota, both of which play an important role in preventing intestinal disease (26). An increasing number of clinical studies have proposed that the composition and the metabolites of gut microbiota were altered in IBD patients (27–29). In addition to gut microbiota, intestinal immune cells also contribute to the intestinal immune responses, especially in IBD. The intestinal immune cells include innate immune cells and adaptive immune cells, which supplement each other and eliminate invading pathogens (30). Moreover, inflammation response is closely related with the immune system. More in detail, the initiation of inflammation is frequently accompanied by the activation of the immune response, which can further activate inflammatory cascade via activating pattern recognition receptors and damage-associated molecular patterns (31, 32). For example, the pathological mechanism of ulcerative proctitis involved multiple factors, including genetic, immune function, and inflammatory responses (33, 34). The pathogenesis of IBDs is complex and involves immune-inflammatory mechanisms. Ginseng has been seen as an important immunomodulator. Specifically, it was found that long-term treatment of ginseng soluble dietary fiber could regulate the secretion levels of immunoglobulins and affect B-cell proliferation, thus further restoring intestinal homeostasis (35).

## 4 Ginseng and ginsenosides

**Figure 1:**
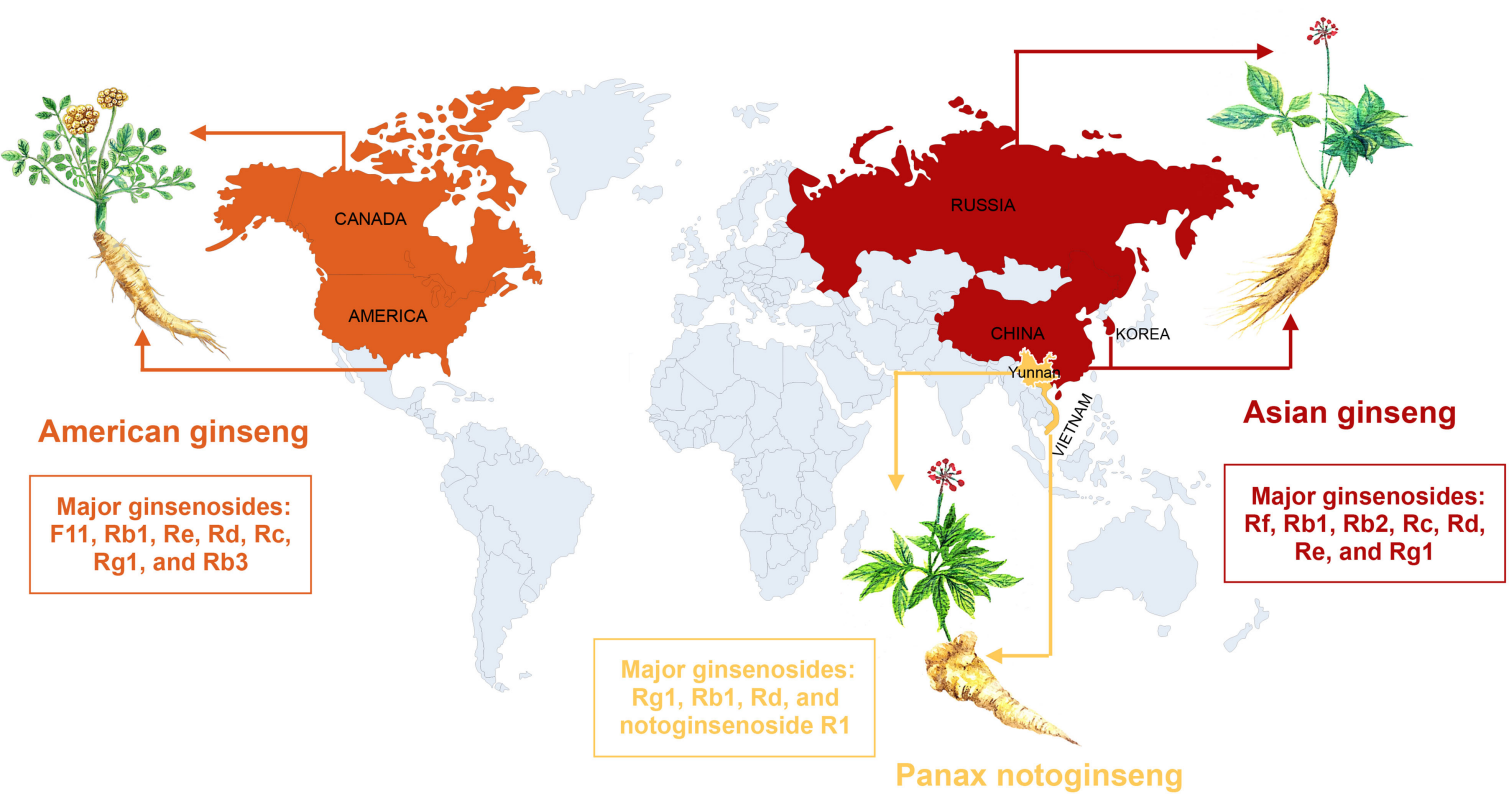
The global distribution of the three most common ginseng herbs, with their major ginsenosides.

Ginseng, the root of *Panax ginseng*, is one of the most frequently used traditional herbal medicine (4). To date, multiple active ingredients have been isolated from ginseng, mainly including ginsenoside, ginseng polysaccharide, and ginseng polypeptide. Among them, ginsenoside is the most abundant and the most studied. The three most common ginseng types included American ginseng, Asian ginseng, and *Panax notoginseng*. We have described their global distribution in **Figure 1**. The content and composition of ginsenosides are varied in different ginseng species. American ginseng is originally grown in the mountain forests of America and Canada and recently has been cultivated in northern China. The major bioactive ginsenosides of American ginseng are F11, Rb1, Re, Rd, Rc, Rg1, and Rb3. Asian ginseng is mainly distributed in Chinese or Korean. The major ginsenosides of Asian ginseng include Rf, Rb1, Rb2, Rc, Rd, Re, and Rg1. *Panax notoginseng*, belonging to the Araliaceae family, is a traditional Chinese medicine, which has been widely used for various diseases, especially cardiovascular diseases. The major ginsenosides of *P. notoginseng* include Rg1, Rb1, Rd, and notoginsenoside R1 (36).

Ginsenosides are the major bio-active component of various ginseng and could be recovered from plant roots, stems, leaves, and flowers. With the recent developments in the extraction and synthetic process, more than 300 ginsenoside monomers have been discovered, such as ginsenoside Rg1, ginsenoside Rb1, ginsenoside Rd, ginsenoside Rf, ginsenoside Re, ginsenoside Rg5, ginsenoside Ra3, and ginsenoside Rd (37). All of them have been confirmed to possess multiple pharmacological effects, including anti-inflammatory (38), anti-aging (39), antioxidant (40), anti-cancer (41), and immuno-modulatory effects (40). On the basis of their chemical structures, ginsenosides can be classed into three types: dammarane, oleanane, and oleanolic acid types. Among them, dammarane can be further divided into protopanaxadiol (PPD), protopanaxatriol (PPT), and ocotillol (OCT) types (42). PPD-type ginsenosides mainly include Rb1, Rb2, Rb3, Rc, Rd, Rg3, and Rh2. PPT-type ginsenosides mainly include Re, Rf, Rg1, Rg2, and Rh1. Previous studies have evaluated that ginsenosides were associated with inflammasome and immune responses (43, 44). For example, it has been demonstrated that ginsenoside Rh1, Rg3, Rg5, Rb1, compound K, and Rg1 were capable of inhabiting inflammatory responses by blocking NLRP3 and NLRP1 (45). Another study further proposed that ginsenosides could enhance the cellular immune function (46). For instance, ginsenoside Rg1 was capable of promoting the proliferation of lymphocytes (47). The functional funding of ginsenosides on inflammasome and immune response provides new insight into the understanding of the molecular mechanisms of ginsenoside-mediated inflammatory and immune actions. More and more pharmacological effects of ginseng and ginsenosides are characterized, especially exhibiting stronger anti-inflammatory and immunomodulatory effects, which have been reckoned as a promising drug for intestinal diseases with great clinical translational potential.

## 5 Effect of ginseng and ginsenosides on intestinal system inflammations

Inflammatory bowel disease is increasingly prevalent in recent years, which greatly affect the gastrointestinal tract function (48). Clinically, despite that a variety of drugs have been used for IBD administration, such as cyclosporin, mesalamine, mercaptopurine, and azathioprine, however, serious side effect and expensive medication cost have greatly limited their clinical application (49). A large number of experiments have shown that ginseng and ginsenosides were capable of effectively relieving the symptoms of IBD through multiple regulatory mechanisms, including regulating the balance of immune cells (50), mediating cytokine expression (51), restoring pathological damage (52), regulating inflammatory signaling pathway (53), and promoting the proliferation of intestinal mucosal epithelium (52). Moreover, in terms of ulcerative colitis (UC) patients, a recent randomized clinical study shows that the rectal co-administration of *P. notoginseng* and Colla Corii Asini suppositories could effectively alleviate their clinical symptom scores and inflammatory factors and improve colon immune function (54). Next, we will discuss all aspects of how ginseng and ginsenosides alleviate IBD in greater detail.

### 5.1 Regulating inflammatory mediators and cytokines

**Figure 2:**
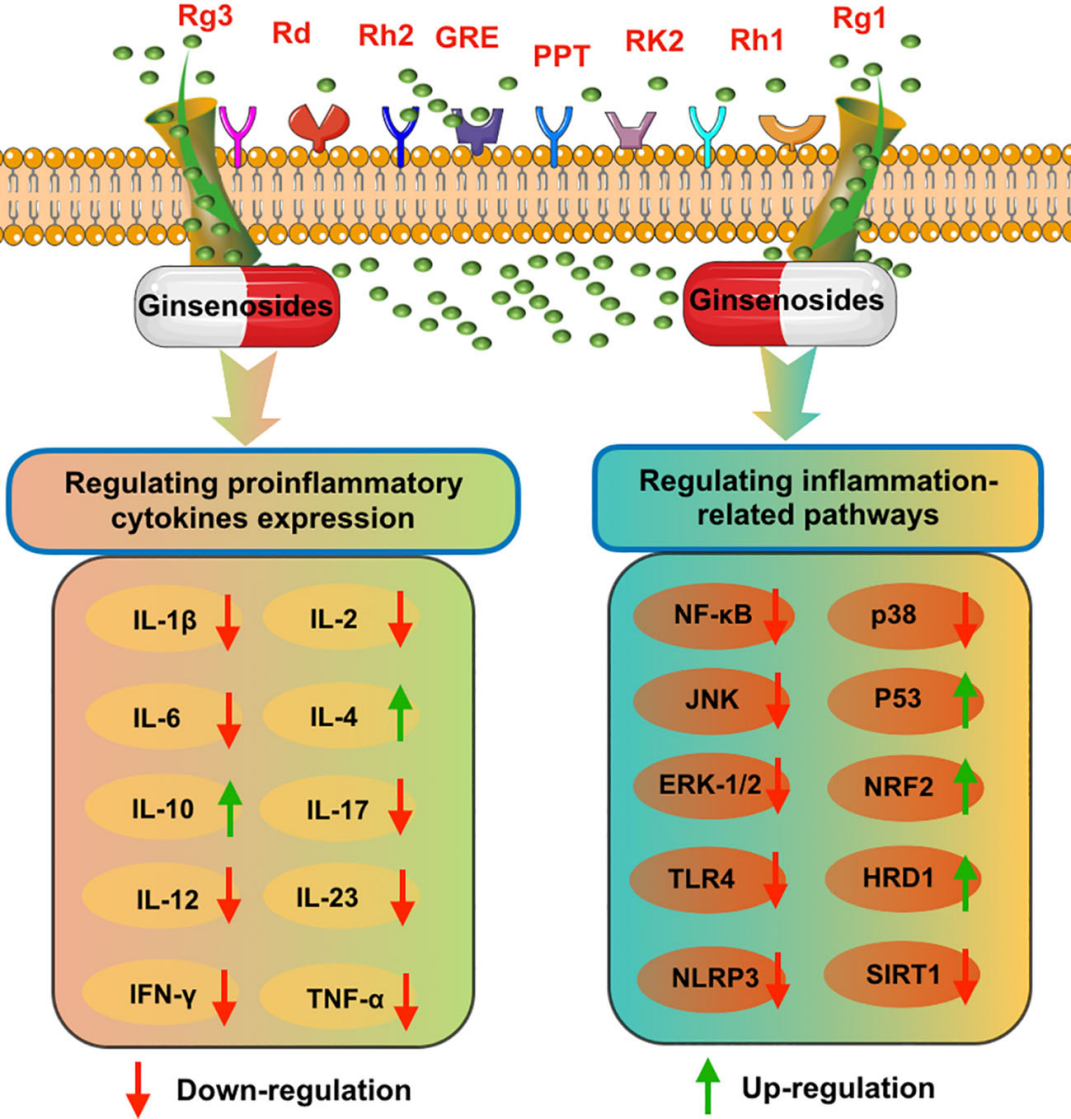
The possible anti-inflammatory mechanisms of various ginsenosides on intestinal system diseases, including inhibiting proinflammatory cytokines expression and blocking inflammation-related pathways.

A growing number of studies have demonstrated that ginseng and its major pharmacologically active components ginsenosides possessed stronger anti-inflammatory effects (55, 56). As shown in **Figure 2**, ginseng and ginsenosides mainly regulate intestinal inflammatory through influencing proinflammatory cytokines expression levels and regulating inflammation-related pathways. Ullah et al. reported that Rg3-enriched Korean Red Ginseng extract could alleviate oxazolone (OXA)-induced UC through suppressing the expression level of NLRP3 and NF-κB (57). Li et al. proposed that ginseng polysaccharides can be used as a promising intervention agent for the prevention of colitis. In a (DSS)-induced rat colitis model, they found that ginseng polysaccharides could effectively alleviate symptoms of colitis and recover the intestinal barrier through downregulating colon inflammatory cytokine levels such as IL-1β, IL-2, IL-6, and IL-17, and blocking the TLR4/MyD88/NF-κB-signaling pathway (58).

In addition, to investigate the protective mechanism of ginsenoside Rd on IBD, 2,4,6-trinitrobenzenesulfonic acid (TNBS) induced colitis rat models were orally administered with ginsenoside Rd for 7 days. The results demonstrated that the inflammatory response was significantly attenuated through downregulating expression levels of proinflammatory cytokines (TNF-α, IL-1β, and IL-6) and blocking the activity of p38 and JNK (59). Moreover, another study also proposed that ginsenosides Rd was able to effectively alleviate DSS colitis in mice through inhibiting proinflammatory cytokines expression (TNF-α, IFN-γ, IL-6, IL-12/23p40, and IL-17A) and inhibiting NF-κB and P38MAPK signaling pathways (60). Ginseng root extract (GRE) also exerted stronger anti-inflammatory and anti-oxidative effects in DSS-induced colitis, which could remarkably inhibit expression levels of inflammatory factors (TNF-α, IL-6, and IL-1β), blocking NF-κB and p62-Nrf2-Keap1 pathways activity, and suppressing the phosphorylation of MAPKs (JNK, ERK-1/2, and p38) (61). Panaxadiol could alleviate DSS induced acute mouse model colitis through suppressing IL-1β secretion and blocking non-canonical caspase-8 inflammasome and MAPKs (62). Huang et al. proposed that ginsenoside Rk2 may be an effective agent in the treatment of UC. They found that ginsenoside Rk2 treatment could block the secretion of proinflammatory cytokines (IL-1β, IL-6, IL-10, and TNF-α) and inactivate ERK/MEK signaling through promoting the dephosphorylation of ERK/MEK and upregulating SIRT1 pathway (63).

In an obesity-induced colonic inflammation-stimulated colitis, in was found that ginsenoside Rk3 could effectively repair the injuries of intestinal epithelial barrier through upregulating the secretion of multiple tight junction proteins and suppressing the expression levels of inflammatory cytokine (TNF-α, IL-1β, and IL-6) and oxidative stress cytokine through blocking the TLR4/NF-κB signaling pathway (64).

Ginsenoside Rb1 (GRb1), one major ginsenoside with multiple pharmacological properties, was capable of effectively alleviating colitis symptoms such as endoplasmic reticulum stress response and fas-related apoptosis through inhibiting inflammatory responses and activating Hrd1 signaling pathway (53). Rh2 has been verified to possess stronger anti-inflammatory and anticancer effects. Based on these characteristics, a recent study found that Rh2 could markedly alleviate various symptoms of (DSS)-induced colitis, mainly including body weight loss, disrupted intestinal barrier functions, colon length shortening, and disease activity index (DAI) scores (65). Luo et al. analyzed the anti-inflammatory and protective effects of *P. notoginseng* saponins (PNS) in IBD both *in vitro* and *in vivo* (DSS-induced colitis mouse model) (66). They found that PNS administration was capable of deregulating secretion levels of proinflammatory cytokines (TNF-α, IL-6, and MCP-1) and blocking the activity of MAPK and NF-κB signaling pathway. Similarly, another study also found that *P. notoginseng* saponins (PNS) could significantly alleviate (DSS)-induced intestinal inflammatory and oxidative stress reactions through upregulating apoptotic cell numbers and blocking PI3K/AKT signaling pathway (67). Moreover, Wang et al. also proposed that *P. notoginseng* could significantly attenuate (DSS-) or iodoacetamide (IA)-induced rat colitis through downregulating serum concentrations of VEGFA isoforms IL-6, and TNF-α, while together upregulating IL-4 and IL-10 (68). In addition, as one main bioactive constituent of *P. notoginseng*, Notoginsenoside R1 also been reported to possess stronger protective effect on IBD, which could effectively relieve the severity of DSS-induced colitis in mice models through suppressing the secretion levels of cytokine and related proinflammatory genes expression (69).

Saba et al. proposed that the co-treatment of red ginseng extract enriched with Rg3 (Rg3-RGE) and *Persicaria tinctoria* could be used for the prevention UC induced by DSS both *in vitro* and *in vivo (* 70). Specifically, in an *in vitro* study, it could inhibit inflammation responses of RAW 264.7 cells through promoting protein kinase and NF-κB pathways. In C57BL/6 mice, this mixture could effectively relieve colitis-related symptoms through exhibiting strong anti-inflammatory effects and suppressing expression levels of NLRP3 inflammasome. In addition, they also proposed that the combination of red ginseng extracts and *Epimedium koreanum* Nakai could alleviate DSS-induced colitis through suppressing protein expression level of proinflammatory cytokines and blocking NF-κB and MAPK pathways (71). Lee et al. found that non-saponin fraction of Korean Red Ginseng possessed stronger intestinal protective effects in a DSS-induced colitis rat model, which could markedly ameliorate gastrointestinal inflammation through suppressing MPO activity, upregulating COX-1 protein expression level, and restoring ZO-1 and occludin secretion to normal levels (72). As one of most commonly used species of ginseng, American ginseng has been discovered to possess multiple protective functions (73). Jin et al. first reported that American ginseng could prevent and treat mouse colitis through suppressing leukocyte activation and subsequent epithelial cell DNA damage (74), promoting inflammatory cell apoptosis, and regulating the activation of P53 (75). Cui et al. found that American ginseng could treat colitis and prevent colon cancer through suppressing the expression levels of ROS and primary proinflammatory markers (76). In addition, their further study proposed that AG and its components also could activate nuclear factor erythroid-2-related factor 2 (Nrf2) pathway, which is involved in colitis progress and closely related with CRC development (77). Consistent with the above results, another study also found that American ginseng could effectively relieve AOM/DSS-induced colon inflammation and suppress tumorigenesis in mouse model through restoring intestinal microbiota function (78).

As an efficient anti-inflammatory agent, Zhu et al. first reported that ginsenoside Rg1 was capable of protecting against DSS-induced mouse colitis through markedly downregulating proinflammatory cytokines secretion (IL-1β and TNF-α) (79). A recent study further discovered that ginsenoside Rg1 could be transformed into 20(S)-protopanaxtriol via ginsenosides Rh1 and F1 through interacting with gut microbiota. Lee et al. found that ginsenosides Rg1, and its major metabolites (Rh1 and 20(S)-protopanaxtriol), all could ameliorate 2,4,6-trinitrobenzene sulfonic acid (TNBS)-induced colitis through suppressing the secretion levels of inflammatory factors (IL-1β, IL-17, and TNF-α), restoring Th17/Treg imbalance, and blocking the binding of LPS to TLR4 on macrophages (80). Another study found that oral administration of probiotic-fermented red ginseng could significantly relieve the symptoms of colitis in (DSS)-induced mouse mode through downregulating the serum levels of inflammatory factors (IL-6 and TNF-α) (81). In addition, one study also found that fermented wild ginseng could relieve the symptoms of colitis in a DSS-induced colitis animal model through inhabiting secretion level of proinflammatory cytokines (IL-1β, IL-6, IL-12, p40, TNF-α, and IFN-γ) and blocking NF-κB signaling pathway (82).

### 5.2 Promoting intestinal mucosal wound healing

In a trinitrobenzenesulfonic-acid-induced rat colitis model, Toyokawa et al. proposed that Daikenchuto and its constituent, ginsenoside Rb1, could remarkably promote intestinal mucosal damage by regulating extracellular-signal-regulated kinase and Rho signaling pathway (83). In addition, another study also demonstrated that *P. notoginseng* administration could promote repair of colonic mucosal injury and microvessels in a (DSS-) or iodoacetamide (IA)-induced rat colitis models through blocking VEGFA isoforms and Rap1GAP/TSP1 pathway (68). Consistent with the above results, Wang et al. also proposed that *P. notoginseng* could repair vascular injury in DSS- and IA-induced colitis animal model through alleviating inflammation responses and oxidative stress (84).

### 5.3 Regulating gut microbiota and metabolism

Intestinal dysbacteriosis has been reckoned as one of the most fundamental factors leading to intestinal diseases, such as IBD, IBS, and CRC (85, 86). Changes in intestinal flora diversity and composition is capable of resulting in imbalances of immune tolerance and dysregulation of intestinal barrier function and upregulating proinflammatory cytokines expression and increasing the incidence of erosion and ulcer (87). Previous studies have found that probiotics supplementation could restore the structure of gut microbiota, thus enhancing intestinal mucosal barrier function and reducing gastrointestinal infection (88). Recent studies further proposed that restoring unbalanced gut microbiota was able to prevent the progression of IBD and intestinal cancer (89). Ginseng and ginsenosides are closely related to the role of gut microbiota, and various non-widespread initial ginsenosides have to be processed and transformed by gut microbiota before they get good biological activity (90, 91). For example, it was found that ginseng polysaccharides could alleviate (DSS)-induced colitis through restoring unbalanced gut microbiota, including increasing the relative abundance of probiotics and, at the same time, imbibing the abundance of pathogenic bacteria (92).

Li et al. also proposed that ginseng polysaccharides exerted a protective effect against (DSS)-induced rat colitis through restoring the diversity and composition of gut microbiota, such as effectively upregulating the relative abundance of Ruminococcus, which also demonstrated that Ruminococcus might be involved in the progression of colitis occurrence (58). The combination treatment of *Zingiber officinale* and *P. ginseng* could ameliorate DSS ulcerative colitis via regulating the abundance of gut microbiome, including upregulating beneficial bacteria such as Muribaculaceae_norank, Lachnospiraceae NK4A136 group, and Akkermansia, and downregulating pathogenic bacteria such as Bacteroides, Parabacteroides, and Desulfovibrio (93). Another study also found that synergistic administration of American ginseng polysaccharide and American ginseng ginsenoside could improve gut microbiota diversity and restore gut microbiota composition, including upregulating the relative abundance of probiotics (Clostridiales, *Bifidobacterium*, and Lachnospiraceae), while together downregulating harmful bacteria (*Escherichia-Shigella* and Peptococcaceae) (94).

In a high-fat diet-induced colitis mice model, it was found that Rk3 could reduce chronic-obesity-induced colitis through alleviating metabolic dysbiosis of gut microbiota and significantly suppressing the ratios of Firmicute/Bacteroidete (64). Prior research has shown that red ginseng could improve the functions of gastrointestinal tract (95). A recent study further proposed that red ginseng could be reckoned as a promising agent for the ulcerative colitis treatment. They found that red ginseng administration significantly relieved the symptoms of trinitro-benzene-sulfonic acid induced ulcerative colitis in a rat model through improving the structure of gut microbiota, including increasing the abundance of probiotics (*Bifidobacterium* and *Lactobacillus*) while inhibiting the growth of some pathogen strains (96).

### 5.4 Preventing colitis-associated colorectal cancer

In addition to ameliorating various symptoms of IBD, some researchers also found that ginseng and ginsenosides also can be used for the prevention of the progression of colitis-associated CRC progression. For example, Wang et al. proposed that oral administration of American ginseng could attenuate AOM/DSS-induced colitis and associated CRC carcinogenesis through downregulating inflammatory factors secretion and restoring the metabolomics and intestinal homeostasis (97). Similarly, Yu et al. also proposed that American ginseng administration was capable of preventing azoxymethane/DSS-induced CRC carcinogenesis through downregulating inflammatory cytokine gene expression (IL-1α, IL-1β, IL-6, IFN-γ, G-CSF, and GM-CSF) (98). Consistent with above results, Poudyal et al. reported that American ginseng possessed stronger anti-inflammatory properties and could prevent azoxymethane/DSS-induced CRC carcinogenesis (99).

In addition, Chen et al. proposed that *P. notoginseng* saponins (PNS) could effectively suppress the progression and development of AOM/DSS-induced colon tumor through regulating the abundance and diversity of gut microbiota, especially obviously increasing the abundance of *Akkermansia* spp., which was negatively associated with the development of CRC (100). It should be noted that *P. notoginseng* saponins (PNS) can be bio-transformed to ginsenoside compound K (GCK) by gut microbiota. Another study found that ginsenoside compound K (GCK) also could inhibit the progression of AOM/DSS-induced colitis-associated CRC by upregulating the relative abundance of *A. muciniphila (* 101).

### 5.5 Alleviate antibiotic-induced diarrhea

Qu et al. proposed that fermented ginseng could alleviate antibiotic-induced diarrhea and colon inflammation through regulating inflammation-related factors such as TNF-α, IL-1β, IL-6, and IL-10 (102). In addition, this study also found that the intestinal flora changes were associated with immune-related factors expression, and the fermented ginseng treatment could recover the alterations of gut microbiota. Similar results were reported by Qu and co-workers. They found that fermented ginseng was able to relieve the symptoms of antibiotic-associated diarrhea through downregulating colon inflammation factors and immune factors (TLR4 and NF-κB) and restoring the gut flora to original intestinal homeostasis (103).

### 5.6 Improving symptoms of irritable bowel syndrome

Irritable bowel syndrome (IBS) is one of the most common gastrointestinal diseases, which affects approximately 10%–20% of the population worldwide, especially in developed countries (104). The symptoms of IBS are experienced as recurrent abdominal pain or discomfort and psychological and physical stressors such as depression or anxiety disorder (105). To date, the pathophysiology of IBS is still unclear (106), mainly including gut microbial dysbiosis, gut–brain axis homeostasis, gut inflammation, and immune dysfunction (107, 108). Ginseng has been confirmed to ameliorate various inflammation responses and help combat depression through suppressing stress (109). An increasing number of studies also demonstrated that ginseng and its major constituent can be reckoned as a promising candidate to treat IBS. For example, Yu et al. proposed that red ginseng (RG) extract significantly improved various symptoms of IBS through downregulating expression level of IL-1β and c-fos, regulating the plasma levels of corticosterone, and restoring the abundance of gut microbiota, including increasing the growth of probiotics microbes (*Lactobacillus johnsonii*, *Lactobacillus reuteri*, and *Parabacteroides goldsteinii) (* 110).

## 6 Effect of ginseng and ginsenosides on intestinal immune disorders

Intestine immune balance is very essential to human body health. Once the balance is broken, it can result in a series of intestinal diseases, such as most common IBD and intestinal tumor (111). Immune dysregulation has been implicated in the pathogenesis of a group of autoimmune diseases. As one of the most common autoimmune diseases, IBD is believed to exhibit a complex and dysregulated response intestinal immune homeostasis, in which the intestinal immune system becomes hyperactive and causes unnecessary impaired integrity of the epithelial barrier (112). As an important immunomodulator, various ginsengs and ginsenosides have been reported to possess a wide range of immuno-modulatory effects, including enhancing host immunity, protecting against various infections and treating immunity-related disorders (113–115).

Specifically, one latest research suggests that, in an oxazolone (OXA)-induced mice UC model, Rg3-enriched Korean Red Ginseng extract was capable of upregulating the number of immune cell subtypes of CD4^+^ T-helper cells, CD19^+^ B-cells, and CD4^+^ and CD25^+^ regulatory T-cells (Tregs), thus significantly improving the colon length and body weight and decreasing disease activity index and histological injury (57). American ginseng has always been recommended as an edible and medicinal functional food use for immunological disorder. One study found that American ginseng and its primary extract (such as polysaccharide and ginsenoside) could significantly reverse the lymphocyte subsets ratio in spleen and peripheral blood and at the same time stimulate CD4^+^T cells and IgA-secreting cells in the small intestine (116). Lu et al. found that *P. notoginseng* saponin (PNS) could significantly relieve (DSS)-induced intestinal colitis through increasing M1 macrophages while decreasing M2 macrophages both in the spleen and colon tissues (67). Kim et al. first reported that Fermented Red Ginseng could obviously relieve 2,4,6-trinitrobenzenesulfonic acid-induced colitis through suppressing macrophage activity and modulating Th1 and Treg cell differentiation (117). In addition to ginseng roots and its various extract, Zhang et al. first reported that ginseng berry extract also could relieve (DSS)-induced colitis through improving the macroscopic appearance of the colon wall, suppressing the activation and number of immune cell (T cells, neutrophils, and CD103^+^CD11c^+^ cDCs), and promoting the migration of CD103^+^CD11c^+^ cDCs (118).

During the most recent years under study, it was found that gut microbiota was involved in regulating mucosal immune balance and host immune response (111, 119). Ginseng and ginsenosides were capable of closely interacting with gut microbiota in the human digestive tract. In addition to directly modulating intestine immune responses, a growing number of studies also reported that ginseng and its various extracts could influence intestinal immune functions via controlling intestinal homeostasis. For example, two previous studies proposed that Korean Red Ginseng-derived polysaccharides could enhancing gut-associated immune functions through increasing the activity of macrophage and promoting Peyer’s patches secretion levels both *in vitro* and in an animal model (120, 121). In addition, Wang et al. reported that oral administration with ginseng polysaccharide was able to relieve lipopolysaccharide induced immunological stress and significantly improve intestinal barrier function (122). Another study found that ginseng-derived small molecule oligopeptides could alleviate irradiation-induced intestinal injury and immune dysfunction through upregulating concentrations of lymphocytes (CD3^+^, CD4^+^, and CD8^+^) and restoring normal baseline intestinal permeability (123). In addition, in another study, Zhu and co-workers found that the intestinal metabolomic effects were significantly different between normal and immunosuppressed rats after ginseng administration (124).

## 7 Conclusions

**Figure 3:**
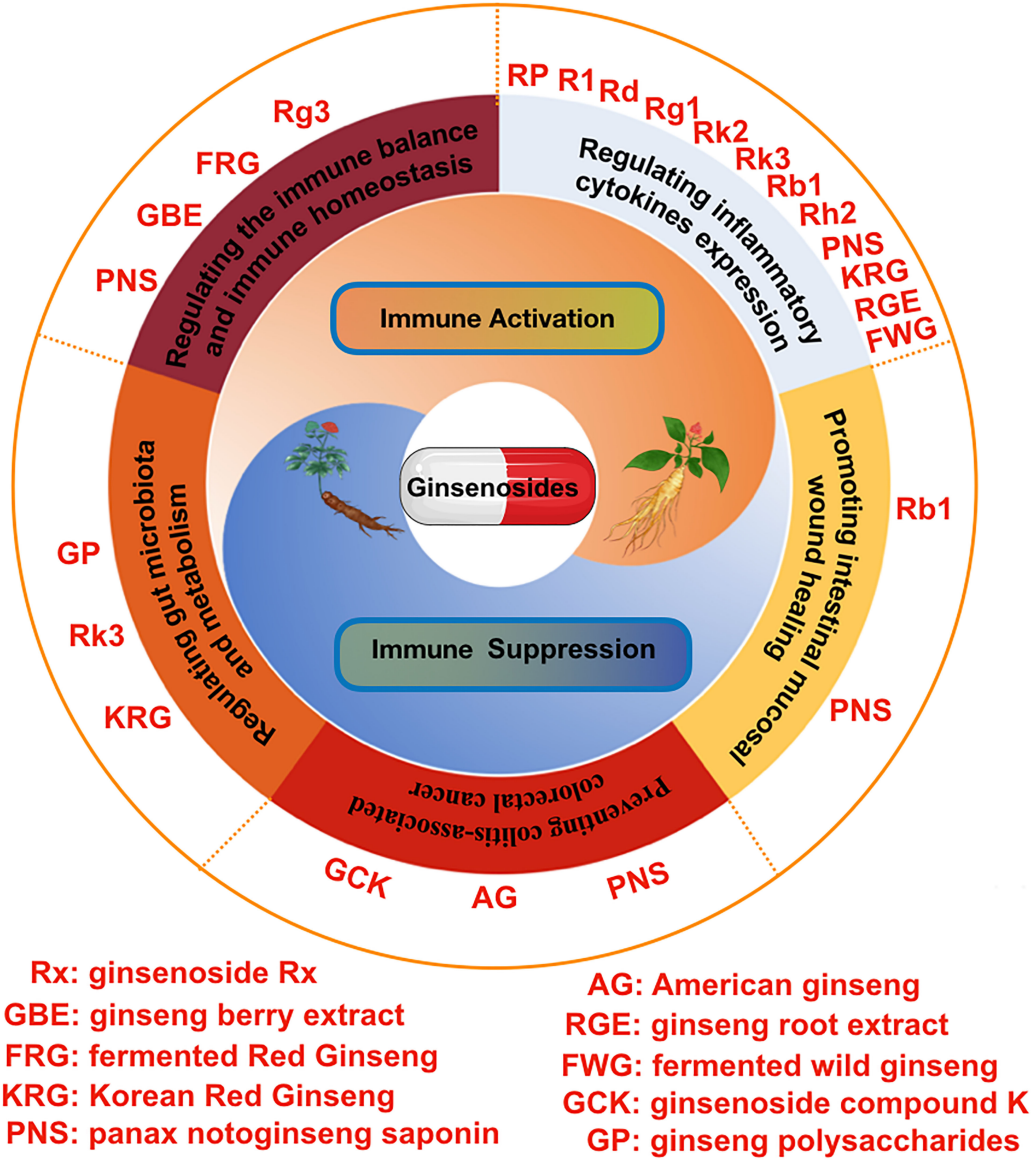
The pharmacological effects of various ginsenosides on intestinal inflammation and the immune system, including regulating inflammatory cytokines expression, regulating the immune balance and immune homeostasis, regulating gut microbiota and metabolism, promoting intestinal mucosal wound healing, and preventing colitis-associated colorectal cancer.

In this article, we systematically summarized the anti-inflammation and immune modulatory effects of various ginseng and ginsenosides in intestinal system (**Table 1**). Intestinal inflammatory imbalance and immune dysfunction may cause a series of intestinal system diseases (125). Ginseng and ginsenosides exert a strong anti-inflammatory and immunomodulatory effect in the intestinal system, and the specific molecular mechanisms were also summarized in this review (**Figure 3**). We can conclude that ginseng and ginsenosides are becoming promising therapeutic options for various gastrointestinal disorders through regulating the immune balance, regulating inflammatory mediators and cytokines, preventing colitis-associated colorectal cancer, regulating gut microbiota and metabolism, alleviating antibiotic-induced diarrhea, and relieving the symptoms of antibiotic-associated diarrhea.

**Table 1:** Anti-inflammation and immune modulatory effects of various ginseng and ginsenosides in intestinal system.

Types	model	effects	Related mechanisms	Refs.
Rg3	OXA-induced mice colitis model	Regulating the immune balance	1. Upregulating the number of immune cell subtypes of CD4^+^ T-helper cells, CD19^+^ B-cells, and CD4^+^ and CD25^+^ regulatory T cells (Tregs) 2. Suppressing the expression level of NLRP3 and NF-κB	(57)
PNS	DSS-induced mice colitis mice model	Regulating the immune balance	1. Increasing M1 macrophages, while decreasing M2 macrophages both in spleen and colon tissues 2. Suppressed activation of the PI3K/AKT signaling pathway	(67)
FRG	Cyclophosphamide-induced immunosuppression and TNBS-induced colitis in mice model	Regulating the immune balance	1. Inhibiting macrophage activation 2. Regulating Th1 and Treg cell differentiation	(117)
GBE	DSS-induced rat colitis model	Regulating the immune balance	1. Inhibited the activation of colon-infiltrating T cells, neutrophils, intestinal CD103^−^CD11c^+^ dendritic cells (cDCs), and macrophages 2. Promoted the migration of CD103^+^CD11c^+^ cDCs and expansion of Foxp3^+^ regulatory T cells in the colons	(118)
RP	DSS-induced rat colitis model	Regulating inflammatory mediators and cytokines	1. Downregulating colon inflammatory cytokine levels such as IL-1β, IL-2, IL-6, and IL-17 2. Blocking the TLR4/MyD88/NF-κB-signaling pathway	(58)
Rd	TNBS induced rat colitis model	Regulating inflammatory mediators and cytokines	1. Downregulating proinflammatory cytokines (TNF-α, IL-1β, and IL-6) expression levels 2. Blocking the activity of p38 and JNK	(59)
Rd	DSS-induced mice colitis model	Regulating inflammatory mediators and cytokines	1. Inhibiting proinflammatory cytokines expression (TNF-α, IFN-γ, IL-6, IL-12/23p40, and IL-17A) 2. Inhibiting NF-κB and P38MAPK signaling pathways	(60)
GRE	DSS-induced mice colitis model	Regulating inflammatory mediators and cytokines	1. Inhibit expression levels of inflammatory factors (TNF-α, IL-6, and IL-1β) 2. Blocking NF-κB and p62-Nrf2-Keap1 pathways activity 3. Suppressing the phosphorylation of MAPKs (JNK, ERK-1/2, and p38)	(61)
0Panaxadiol	DSS-induced mice colitis model	Regulating inflammatory mediators and cytokines	1. Suppressing IL-1β secretion 2. Blocking non-canonical caspase-8 inflammasome and MAPKs	(62)
Rk2	Caco-2 cells and THP-1 cells	Regulating inflammatory mediators and cytokines	1. Block the secretion of pro-inflammatory cytokines (IL-1β, IL-6, IL-10, and TNF-α) 2. Inactivating ERK/MEK signaling through promoting the dephosphorylation of ERK/MEK and upregulating SIRT1 pathway	(63)
Rk3	Obesity-induced colonic inflammation-stimulated colitis in mice model	Regulating inflammatory mediators and cytokines	1. Upregulating the secretion of multiple tight junction proteins 2. Suppressing the expression levels of inflammatory cytokine (TNF-α, IL-1β, and IL-6) and oxidative stress cytokine 3. Blocking the TLR4/NF-κB signaling pathway	(64)
Rb1	DSS-induced mice colitis model	Regulating inflammatory mediators and cytokines	1. Inhibiting inflammatory responses 2. Activating Hrd1 signaling pathway	(53)
Rh2	DSS-induced mice colitis model	Regulating inflammatory mediators and cytokines	1. Inducing reactive oxygen species (ROS) 2. Activating NF-kB survival pathway 3. Activating p53 pathway	(65)
PNS	DSS-induced mice colitis model	Regulating inflammatory mediators and cytokines	1. Regulating secretion levels of proinflammatory. cytokines (TNF-α, IL-6, and MCP-1) 2. Blocking the activity of MAPK and NF-κB signaling pathway	(66)
PNS	DSS-induced rat colitis model	Regulating inflammatory mediators and cytokines	1. Upregulating apoptotic cell numbers and blocking PI3K/AKT signaling pathway	(67)
PNS	DSS- or IA-induced rat colitis model	Regulating inflammatory mediators and cytokines	1. Downregulating serum concentrations of VEGFA isoforms IL-6, and TNF-α 2. Upregulating IL-4 and IL-10	(68)
R1	DSS-induced mice colitis model	Regulating inflammatory mediators and cytokines	1. Suppressing the secretion levels of cytokine and. related proinflammatory genes expression	(69)
Rg3-RGE	DSS-induced mice colitis model	Regulating inflammatory mediators and cytokines	1. Inhibit inflammation responses of RAW 264.7 cells through promoting protein kinase and NF-κB pathways 2. Exhibiting strong anti-inflammatory effects 3. Suppressing expression levels of NLRP3 inflammasome 4. Blocking NF-κB and MAPK pathways	(70)
KRG	DSS-induced rat colitis model	Regulating inflammatory mediators and cytokines	1. Ameliorating gastrointestinal inflammation through suppressing MPO activity 2. Upregulating COX-1 protein expression level 3. Restoring ZO-1 and occludin secretion to normal. levels	(72)
AG	DSS-induced mice colitis model	Regulating inflammatory mediators and cytokines	1. Suppressing leukocyte activation and subsequent epithelial cell DNA damage	(74)
AG	DSS-induced mice colitis model	Regulating inflammatory mediators and cytokines	1. Promoting inflammatory cell apoptosis 2. Regulating the activation of P53	(75)
AG	AOM/DSS -induced mice colitis model	Regulating inflammatory mediators and cytokines	1. Suppressing the expression levels of ROS and. primary proinflammatory markers 1. Activating nuclear factor erythroid-2-related factor 2 (Nrf2) pathway	(76)
AG	High fat diet-fed AOM/DSS -induced mice colitis model	Regulating inflammatory mediators and cytokines	1. Restoring intestinal microbiota function	(78)
Rg1	DSS-induced mice colitis model	Regulating inflammatory mediators and cytokines	1. Downregulating proinflammatory cytokines secretion (IL-1β and TNF-α)	(79)
Rg1	TNBS-induced mice colitis model	Regulating inflammatory mediators and cytokines	1. Suppressing the secretion levels of inflammatory. factors (IL-1β, IL-17, and TNF-α) 2. Restoring Th17/Treg imbalance 3. Blocking the binding of LPS to TLR4 on. macrophages	(80)
PFRG	DSS-induced mice colitis model	Regulating inflammatory mediators and cytokines	1. Downregulating the serum levels of inflammatory factors (IL-6 and TNF-α)	(81)
FWG	DSS-induced mice colitis model	Regulating inflammatory mediators and cytokines	1. Inhabiting secretion level of pro-inflammatory cytokines (IL-1β, IL-6, IL-12p40, TNF-α, and IFN-γ) 2. Blocking NF-κB signaling pathway	(82)
Rb1	TNBS-induced rat colitis model	Promoting intestinal mucosal wound healing	1. Regulating extracellular signal-regulated kinase and. Rho signaling pathway	(83)
PNS	DSS- or IA-induced rat colitis models	Promoting intestinal mucosal wound healing	1. Blocking VEGFA isoforms and Rap1GAP/TSP1 pathway	(68)
PNS	DSS- or IA- induced rat colitis models	Promoting intestinal mucosal wound healing	1. Alleviating inflammation responses and oxidative stress 2. Downregulating proinflammatory cytokine 3. Regulating apoptotic related gene expression	(84)
GP	DSS-induced rat colitis model	Regulating gut microbiota and metabolism	1. Increasing the relative abundance of probiotics 2. Imbibing the abundance of pathogenic bacteria	(92)
GP	DSS-induced rat colitis model	Regulating gut microbiota and metabolism	1. Restoring the diversity and composition of gut. microbiota 1. Upregulating the relative abundance of *Ruminococcus*	(58)
PG	DSS-induced rat colitis model	Regulating gut microbiota and metabolism	1. Upregulating beneficial bacteria such as Muribaculaceae_norank, Lachnospiraceae NK4A136 group and Akkermansia 2. Downregulating pathogenic bacteria such as Bacteroides, Parabacteroides and Desulfovibrio	(93)
GP	CTX-induced intestinal immune disorders and gut barrier dysfunctions in mice model	Regulating gut microbiota and metabolism	1. Upregulating the relative abundance of probiotics (Clostridiales, *Bifidobacterium*, and Lachnospiraceae) 2. Regulating mitochondrial−related pathway 3. Downregulating harmful bacteria (*Escherichia*-*Shigella* and Peptococcaceae)	(94)
Rk3	High-fat diet-induced mice colitis model	Regulating gut microbiota and metabolism	1. Alleviating metabolic dysbiosis of gut microbiota 2. Suppressing the ratios of Firmicute/Bacteroidete	(64)
KRG	TBSA-induced rat ulcerative colitis model	Regulating gut microbiota and metabolism	1. Increasing the abundance of probiotics (*Bifidobacterium* and *Lactobacillus*) 2. Inhibiting the growth of some pathogen strains	(96)
AG	AOM/DSS-induced mice colitis model	Preventing colitis-associated colorectal cancer	1. Downregulating inflammatory factors secretion 2. Restoring the metabolomics and intestinal homeostasis	(97)
AG	AOM/DSS-induced mice colitis model	Preventing colitis-associated colorectal cancer	1. Downregulating inflammatory cytokine gene expression (IL-1α, IL-1β, IL-6, IFN-γ, G-CSF, and GM-CSF)	(98)
PNS	AOM/DSS-induced colitis-associated CRC model	Preventing colitis-associated colorectal cancer	1. Regulating the abundance and diversity of gut microbiota 2. Increasing the abundance of *Akkermansia* spp.	(100)
GCK	AOM/DSS-induced colitis-associated CRC model	Preventing colitis-associated colorectal cancer	1. Upregulating the relative abundance of *A. muciniphila* 2. Inducing cytoplasmic Ca^2+^	(101)
FG	Antibiotic-rat diarrhea model	Alleviating antibiotic-induced diarrhea and colon inflammation	1. Regulating inflammation-related factors such as TNF-α, IL-1β, IL-6, and IL-10 2. Recovering the alterations of gut microbiota returned to normal level	(102)
FG	Antibiotic- induced rat diarrhea model	Relieve the symptoms of antibiotic-associated diarrhea	1. Downregulating colon inflammation factors and immune factors (TLR4 and NF-κB) 2. Restoring the gut flora to original intestinal homeostasis	(103)
KRG	Intracolonic-zymosan-induced post-infectious human IBS-like symptoms mice model	Improving various symptoms of irritable bowel syndrome	1. Downregulating expression level of IL-1β and c-fos 2. Regulating the plasma levels of corticosterone 3. Restoring the abundance of gut microbiota 4. Increasing the growth of probiotics microbes (*Lactobacillus johnsonii*, *Lactobacillus reuteri*, and *Parabacteroides goldsteinii*)	(110)
KRGP	RAW264.7 cells	Improving intestinal immune disorders	1. Increasing the activity of macrophage and promoting Peyer’s patches secretion levels both *in vitro* and in animal model	(120)
GP	Irradiation-induced immune dysfunction and subsequent intestinal injury both *in vitro* and *in vivo* models	Improving intestinal immune disorders	1. Upregulating concentrations of lymphocytes (CD3^+^, CD4^+^ and CD8^+^) 2. Restoring normal baseline intestinal permeability	(123)

Rg3, ginsenoside Rg3; PNS, Panax notoginseng saponin; FRG, fermented Red Ginseng; GBE, ginseng berry extract; GP, ginseng polysaccharides; Rd, ginsenoside Rd; GRE, ginseng root extract; Rk2, ginsenoside Rk2; Rk3, ginsenoside Rk3; Rb1, ginsenoside Rb1; Rh2, ginsenoside Rh2; R1, ginsenoside R1; Rg3-RGE, red ginseng extract enriched with Rg3; KRG, Korean Red Ginseng; AG, American ginseng; Rg1, ginsenoside Rg1; PFRG, probiotic-fermented red ginseng; FWG, fermented wild ginseng; PG, Panax ginseng; GCK, ginsenoside compound K; FG, fermented ginseng; KRGP, Korea red ginseng-derived polysaccharides; OXA, oxazolone; DSS, dextran sulfate sodium; TNBS, 2,4,6-trinitrobenzenesulfonic acid; IA, iodoacetamide; AOM, azoxymethane; CTX, Cyclophosphamide; TBSA, trinitro-benzene-sulfonic acid; CRC, colorectal cancer.

## Author contributions

LZ: Writing – original draft, Supervision, Conceptualization. TZ: Writing – original draft, Investigation, Conceptualization. KZ: Writing – original draft, Supervision, Project administration, Conceptualization.
